# Intelligent systemic microbial monitoring method for fresh food supply chain safety

**DOI:** 10.3389/fmicb.2026.1974318

**Published:** 2026-10-28

**Authors:** Qing Tian

**Affiliations:** School of Modern Logistics, Shanxi Vocational University of Engineering Technology, Jinzhong, Shanxi, China

**Keywords:** availability-aware learning, ForwardSpectralProbabilistic, fresh food supply chain safety, Harmonic State Projection, heterogeneous data, microbial monitoring, spectral consistency, temporal consistency

## Abstract

**Introduction:**

Microbial safety monitoring in fresh-food systems increasingly relies on heterogeneous information sources, including hyperspectral measurements, microscopy images, structured environmental variables, and temporally related observations. These information sources are not uniformly available across monitoring scenarios, making it inappropriate to impose the same temporal, spectral, or contextual assumptions on every sample. This study proposes the ForwardSpectralProbabilistic (FSP) framework for microbial classification and pathogen-risk prediction under heterogeneous sensing conditions.

**Methods:**

FSP employs modality-specific encoders to map spectral, image, and structured observations to a common latent dimensionality while preserving their native input representations. Contextual information is incorporated through gated residual fusion when environmental, operational, or acquisition metadata are available. The Forward Consistency Projector generates temporally aligned latent evidence only for observations with a valid predecessor target correspondence, whereas the Spectral Consistency Mapper operates only on measurements with a native spectral axis. The Probabilistic Mixture Integrator then combines the available current, temporal, and spectral representations through availability-normalized learned weights. Harmonic State Projection (HSP) is further introduced as a structured regularization strategy that constrains temporal projection consistency and preserves relationships among low-frequency spectral components without constructing unavailable modalities. The framework is evaluated independently on four publicly accessible microbial-safety datasets covering Salmonella serovar classification from hyperspectral measurements, microscopy-based bacterial classification, poultry Salmonella-Campylobacter risk prediction, and agricultural-water microbial marker prediction. The experiments use modality-matched comparison methods, grouped or stratified data partitioning, and repeated random seeds.

**Results and discussion:**

The descriptive results show that FSP is competitive across heterogeneous tasks, while its strongest observed performance is not obtained uniformly for every metric and dataset. Spectral-consistency and harmonic-relation modeling provide the clearest gains for native hyperspectral measurements, meteorological context and temporally aligned evidence contribute to the structured poultry-risk task, and acquisition metadata improve robustness under microscopy domain shift. Modality-aware ablation experiments further indicate that spectral, temporal, and contextual mechanisms provide complementary contributions only when the corresponding information is available. FSP provides an availability-aware formulation for microbial monitoring that maintains a consistent prediction framework without imposing artificial spectral or temporal structure on heterogeneous food-safety data.

## 1 Introduction

Microbial contamination remains a persistent challenge for food safety because biological hazards may arise during production, processing, storage, transportation, or retail, while modern food-supply systems increasingly require rapid and data-informed responses to emerging risks (Singh, 2025). The transition from predominantly retrospective inspection toward predictive and early-warning food-safety management has consequently increased interest in artificial intelligence (AI), big data, and sensor-assisted monitoring (Mu et al., 2024). For fresh food products in particular, rapid optical sensing combined with machine learning has demonstrated the potential to estimate pathogen-related conditions without relying exclusively on lengthy conventional laboratory workflows (Sharma and Tharani, 2024).

Recent advances further indicate that microbial-safety information can be represented through substantially different sensing and data modalities. Hyperspectral imaging combined with machine-learning models has enabled rapid and non-destructive discrimination of foodborne pathogens from high-dimensional wavelength measurements (Feng et al., 2025). Time-dependent pathogen sensing has also been explored using machine-learning-enabled chromogenic measurements, where temporal sensor responses can help distinguish pathogenic bacteria in the presence of background microflora (Jia et al., 2024). Beyond direct microbial sensing, environmental and operational variables can provide complementary predictive information, as meteorological conditions and farm-management characteristics have been associated with variations in microbial levels (Qian et al., 2025). Together, these developments suggest that microbial monitoring is increasingly a heterogeneous-data problem involving spectral measurements, microscopy images, environmental variables, acquisition context, and temporal information rather than a single uniform input representation.

The reliability of AI-based food-safety systems nevertheless depends strongly on the quality, representativeness, harmonization, and availability of the underlying data (Dimitrakopoulou and Garre, 2025). This issue becomes particularly important when different monitoring scenarios provide different subsets of information. A hyperspectral experiment may contain a native wavelength axis without longitudinal correspondence, a microscopy experiment may provide image observations together with acquisition metadata, and a regulatory or environmental dataset may contain structured predictors with contextual or temporally ordered information. Imposing spectral, temporal, or contextual assumptions uniformly across these settings would introduce structure that is not supported by the corresponding measurements. Conversely, treating every sensing configuration as an entirely independent methodological problem limits the ability to maintain a consistent prediction formulation across microbial-monitoring tasks. A suitable framework should therefore preserve modality-specific information while activating temporal, spectral, or contextual mechanisms only when the required evidence is genuinely available.

To address this challenge, this study proposes the ForwardSpectralProbabilistic (FSP) framework for microbial classification and pathogen-risk prediction under heterogeneous sensing conditions. Rather than converting different raw inputs into an artificial common modality, FSP employs modality-specific encoders for hyperspectral, microscopy-image, and structured observations and maps them to a common *d*-dimensional latent interface within each dataset-specific experiment. Environmental, operational, or acquisition context is incorporated through availability-aware gated residual fusion when such information is present. The Forward Consistency Projector generates temporally aligned latent evidence only when a valid predecessor-target correspondence can be defined, whereas the Spectral Consistency Mapper operates only on observations with a native spectral axis. The Probabilistic Mixture Integrator then combines the available current, temporal, and spectral representations through learned availability-normalized weights. Harmonic State Projection (HSP) is introduced as a structured regularization strategy that constrains temporal projection consistency and preserves harmonic relationships in native spectral measurements without constructing unavailable modalities or artificial temporal links.

The main contributions of this study are summarized as follows:

- An availability-aware formulation is introduced for heterogeneous microbial monitoring with hyperspectral, microscopy-image, and structured observations. The formulation preserves native input modalities, separates primary and contextual representations, and excludes unavailable temporal or spectral information rather than replacing it with artificially generated evidence.
- An implementable FSP architecture is developed using modality-specific encoders, contextual gated fusion, the Forward Consistency Projector, the Spectral Consistency Mapper, and the Probabilistic Mixture Integrator. The accompanying HSP strategy provides explicit temporal-projection, spectral-consistency, and harmonic-relation objectives that are activated only for observations supporting the corresponding information source.
- The framework is evaluated independently on four publicly accessible microbial-safety datasets covering hyperspectral Salmonella serovar classification, microscopy-based bacterial classification, poultry pathogen-risk prediction, and agricultural-water microbial-marker prediction. The evaluation uses modality-matched comparison methods, grouped or stratified partitioning, repeated random seeds, explicitly defined AUC calculations, and out-of-distribution evaluation where applicable.
- A modality-aware ablation analysis is conducted so that only components applicable to a given dataset are removed. The experiments separately examine spectral-consistency and harmonic-relation objectives, temporal evidence, contextual information, gated fusion, and learned evidence integration, thereby distinguishing their contributions under different information availability conditions.

## 2 Related work

### 2.1 AI-enabled microbial detection and microscopy

Recent advances in optical sensing and artificial intelligence have enabled microbial detection systems to move beyond manual visual inspection toward automated extraction of spectral, morphological, and spatial characteristics. Hyperspectral microscopic imaging has been combined with deep learning to distinguish viable and non-viable foodborne bacteria by jointly exploiting spectral signatures and morphological information (Park et al., 2023). More recent spatial spectral architectures directly process bacterial hyperspectral cubes and learn discriminative three-dimensional features without relying entirely on manually selected wavelengths or handcrafted descriptors (Kang et al., 2024). Microscopy images have also become an important source of information for rapid bacterial identification. Deep convolutional models have been evaluated for automated recognition of multiple common foodborne bacterial species from microscopic images, demonstrating the feasibility of reducing reliance on manual morphology-based identification (Chen et al., 2024). Microcolony-level detection has further been extended to complex food matrices, where models must distinguish live bacterial structures from visually similar food debris rather than from clean laboratory backgrounds alone (Park et al., 2025). Temporal acquisition has recently been incorporated into microscopy-based pathogen analysis. Time-stamped microscopy images collected during early incubation have been used for instance-level segmentation of *E. coli* and *Salmonella* under both clean and food-associated background conditions, showing that incubation stage and background variation affect early microbial detection (Koirala et al., 2025). Architectural studies based on YOLOv8 have also shown that bacterial microcolony detection is sensitive to backbone selection and multi-scale feature-fusion design, particularly for small and low-contrast microbial targets (Rababa et al., 2026). These studies demonstrate that wavelength information, microscopic morphology, acquisition conditions, and time-related measurements can each provide useful microbial evidence. However, most existing approaches are developed for a particular sensing modality and prediction task. Time-stamped image collections also do not necessarily imply the sample-level predecessor target correspondence required by the temporal mechanism in FSP. The proposed framework therefore preserves modality-specific feature extraction and introduces temporal or spectral processing only when the required information structure is explicitly supported by the data.

### 2.2 Environmental sensing and supply-chain monitoring

Microbial risk during food production and distribution is influenced not only by direct pathogen measurements but also by environmental and operational conditions that change during storage and transportation. Smart packaging systems have incorporated disposable wireless gas sensors and near-field communication to support continuous in-package monitoring of food spoilage under different storage conditions (Naik et al., 2024). Integrated cold-chain platforms have also combined IoT sensing, digital traceability, and machine-learning-based quality evaluation to associate environmental measurements with changes in transported food products (Feng et al., 2024). Sensor-derived temporal information is increasingly used as an input to data-driven prediction rather than solely as a traceability record. Time temperature measurements together with initial product characteristics have been used to forecast changes in strawberry marketability and quality during storage and transportation (Elashmawy et al., 2025). Such work illustrates the value of preserving temporal structure when repeated measurements are genuinely linked to the same monitored process. Environmental conditions can also influence microbial persistence and cross-contamination directly. Controlled cold-chain experiments have shown that the survival and transfer behavior of *E. coli* O157:H7 varies with packaging material, temperature, contact conditions, and exposure time and that these non-linear relationships can be modeled using machine-learning methods (Wang et al., 2025). These findings support the use of environmental measurements as contextual predictors when such information is available. The present study does not treat sensing, communication, or traceability infrastructure itself as part of the FSP prediction model. Instead, environmental, operational, and acquisition variables are represented as contextual evidence and incorporated through gated fusion with the primary microbial representation. This distinction permits contextual information to contribute to prediction without assuming that each benchmark contains a complete longitudinal trace spanning production, processing, transportation, storage, and retail.

### 2.3 Predictive microbial risk assessment

Predictive microbial risk modeling seeks to estimate contamination or pathogen-related outcomes using microbial, environmental, spatial, or temporal information before retrospective analysis alone can support operational decisions. Environmental prediction has been investigated for agricultural water used in fresh-produce production. Conditional random forest and regression models have been evaluated for predicting general and human-associated fecal contamination in irrigation ponds using microbial and environmental predictors, while their performance can deteriorate when models are transferred across locations or sampling periods (Hofstetter et al., 2024). This limitation highlights the distinction between predictive associations learned within a particular dataset and relationships that generalize to new environments. Food-safety surveillance data have also been used to construct machine-learning early-warning systems. Spatiotemporal surveillance variables and food-safety inspection information have been integrated with tree-based models to anticipate regional patterns of human salmonellosis (Garcia-Vozmediano et al., 2024). Lagged climatic variables have further been incorporated into poultry contamination prediction, indicating that environmental conditions preceding sampling can contain information relevant to subsequent *Salmonella* risk (Sylla et al., 2026). Temporal and environmental information has also begun to appear directly in AI-based pathogen imaging. An explainable framework for co-contaminated food-pathogen detection has combined microscopic evidence with temporal and environmental information, providing a close conceptual connection between microbial imaging and context-aware prediction (AlSobeh et al., 2025). Nevertheless, this setting remains centered on a specific microscopic pathogen-detection problem, whereas heterogeneous food-safety benchmarks may provide native spectra, microscopy images, structured contextual variables, valid temporal relationships, or only a subset of these information sources. FSP addresses this heterogeneity through availability-aware computation rather than through joint multimodal training that assumes every observation contains the same information. Modality-specific encoders first map native observations to a common latent dimensionality, while contextual variables are incorporated through gated residual fusion when available. The Forward Consistency Projector generates temporally aligned evidence only when a defensible predecessor target relationship exists, whereas the Spectral Consistency Mapper operates only on measurements with a native wavelength axis. Harmonic State Projection provides temporal-projection and harmonic-relation regularization during training, while the Probabilistic Mixture Integrator combines only the current, temporal, and spectral latent representations that are available for an observation. This formulation therefore differs from modality-specific detection and early-warning approaches by maintaining a consistent prediction framework without requiring all sensing modalities or auxiliary information to coexist in every microbial-monitoring task.

## 3 Method

### 3.1 Overview

This section presents the Intelligent Systemic Microbial Monitoring Method, which is designed for microbial classification and pathogen-risk prediction under heterogeneous food-safety monitoring conditions. The method is built upon the ForwardSpectralProbabilistic (FSP) framework and accommodates different forms of microbial evidence, including hyperspectral measurements, microscopy images, and structured environmental or microbiological observations. Rather than assuming that all samples contain the same sensing modalities or auxiliary information, FSP preserves the native structure of each input modality and activates contextual, temporal, and spectral components only when the corresponding evidence is available. The datasets considered in this study are trained and evaluated independently; the shared latent representation therefore refers to a common *d*-dimensional representation interface within the framework rather than to joint cross-dataset training.

Section 3.2 formalizes the heterogeneous microbial monitoring problem and defines the representations used throughout the framework. Modality-specific encoders map hyperspectral, image, and structured observations into a common latent dimensionality, while auxiliary environmental, operational, or acquisition variables are represented separately as contextual information. The formulation further defines availability indicators for contextual, temporal, and spectral evidence and introduces the corresponding task, temporal-consistency, spectral-consistency, and harmonic-relation objectives. Missing information is excluded through availability-aware masking rather than replaced by artificially generated modalities or temporal relationships. Section 3.3 specifies the forward computation of FSP. The architecture first performs modality-specific feature extraction and availability-aware contextual gated fusion to obtain the current latent state. When a valid temporal predecessor exists, the Forward Consistency Projector generates a temporally aligned latent representation for the target observation. For measurements containing a native spectral axis, the Spectral Consistency Mapper estimates a harmonic descriptor and maps the resulting spectral evidence back into the shared latent dimensionality. The Probabilistic Mixture Integrator then assigns learned weights only to the available current, temporal, and spectral representations and produces an integrated latent state, which is converted into class or pathogen-risk probabilities by a dataset-specific prediction head. Section 3.4 introduces Harmonic State Projection (HSP) as a structured regularization strategy embedded within FSP rather than as an independent prediction module. HSP contains two complementary constraints. The temporal projection constraint encourages agreement between a forward-projected latent state and its observed target representation when a valid temporal correspondence is present. The harmonic relation constraint preserves both coefficient-level spectral information and the relative structure among retained low-frequency components when native spectral measurements are available. These constraints are activated independently according to data availability and contribute to training without altering the modality requirements of the prediction process.

Accordingly, the proposed framework separates native-modality feature extraction, contextual representation, structured auxiliary regularization, and probabilistic evidence integration. Static image or structured observations can be processed without constructing artificial temporal or spectral information, whereas temporally linked or hyperspectral observations can additionally exploit the corresponding consistency constraints. This conditional formulation maintains a consistent prediction pipeline across heterogeneous microbial-monitoring tasks while preserving an explicit correspondence between the available data, model components, training objectives, and inference procedure.

### 3.2 Preliminaries

This subsection formalizes microbial classification and pathogen-risk prediction under heterogeneous sensing conditions. The experimental datasets considered in this study contain different forms of observations, including hyperspectral measurements, microscopy images, and structured environmental or microbiological variables. These datasets are evaluated independently and are not merged at the sample level. A modality-specific encoder first maps each primary observation into a shared latent space, after which contextual, temporal, and spectral information is incorporated only when the corresponding evidence is available (Equation 1).

Let

$$\begin{array}{l} D^{\left(r\right)}={\left\{\left(x_{j}^{\left(r\right)},e_{j}^{\left(r\right)},\tau_{j}^{\left(r\right)},y_{j}^{\left(r\right)}\right)\right\}}_{j=1}^{N_{r}}, \end{array}$$ (1)

denote the *r*-th dataset, where *N*_*r*_ is the number of observations. Here, $x_{j}^{\left(r\right)}$ denotes the primary observation, $e_{j}^{\left(r\right)}$ denotes auxiliary environmental, operational, or acquisition context when such information is available, $\tau_{j}^{\left(r\right)}$ denotes the sampling time or acquisition order when a defensible temporal correspondence exists, and $y_{j}^{\left(r\right)}$ denotes the prediction target. Depending on the dataset, $x_{j}^{\left(r\right)}$ may be a hyperspectral signature, a microscopy image, or a structured vector of microbiological and environmental measurements. When no auxiliary context is defined for an observation, $e_{j}^{\left(r\right)}$ is treated as unavailable rather than as an additional synthetic input.

For a *C*_*r*_-class classification task (Equation 2),

$$\begin{array}{l} y_{j}^{\left(r\right)}\in \left\{1,\ldots ,C_{r}\right\}, \end{array}$$ (2)

whereas for a binary pathogen-risk or microbial-marker prediction task (Equation 3),

$$\begin{array}{l} y_{j}^{\left(r\right)}\in \left\{0,1\right\}. \end{array}$$ (3)

To preserve the native structure of heterogeneous raw measurements, a modality-specific encoder is introduced (Equation 4):

$$\begin{array}{l} z_{j}^{\left(r\right)}=E_{m_{r}}\left(x_{j}^{\left(r\right)};\theta_{m_{r}}\right),\;\;z_{j}^{\left(r\right)}\in {\mathbb{R}}^{d}, \end{array}$$ (4)

where Equation 5

$$\begin{array}{l} m_{r}\in \left\{\text{spec},\text{img},\text{tab}\right\} \end{array}$$ (5)

denotes the input modality, θ_*m*_*r*__ denotes the parameters of the corresponding encoder, and *d* is the dimensionality of the shared latent space. The spectral encoder operates along the ordered wavelength axis, the image encoder extracts spatial and morphological information from microscopy observations, and the tabular encoder processes structured numerical and categorical variables. The implementation of the modality-specific encoders is described in Section 3.3.

When auxiliary contextual variables are available, they are independently encoded as Equation 6

$$\begin{array}{l} c_{j}^{\left(r\right)}=E_{c}\left(e_{j}^{\left(r\right)};\theta_{c}\right)\in {\mathbb{R}}^{d}. \end{array}$$ (6)

Let $a_{j,\text{ctx}}^{\left(r\right)}\in \left\{0,1\right\}$ indicate whether contextual information is available. The fused current-state representation is defined as Equation 7

$$\begin{array}{l} h_{j}^{\left(r\right)}=F_{\text{fuse}}\left(z_{j}^{\left(r\right)},c_{j}^{\left(r\right)};a_{j,\text{ctx}}^{\left(r\right)}\right),\;\;h_{j}^{\left(r\right)}\in {\mathbb{R}}^{d}, \end{array}$$ (7)

where $F_{\text{fuse}}$ denotes the availability-aware contextual fusion operator. When $a_{j,\text{ctx}}^{\left(r\right)}=0$, the contextual contribution is masked and the fused representation reduces to the primary latent representation. The specific gated fusion mechanism is given in Section 3.3.

Temporal modeling is used only when two observations possess a genuine temporal correspondence. Let $h_{j,t}^{\left(r\right)}$ and $h_{j,t+1}^{\left(r\right)}$ denote two linked latent states separated by Equation 8

$$\begin{array}{l} \Delta \tau_{j,t}=\tau_{j,t+1}^{\left(r\right)}-\tau_{j,t}^{\left(r\right)}. \end{array}$$ (8)

The Forward Consistency Projector estimates the latent representation aligned with the subsequent observation (Equation 9):

$$\begin{array}{l} {\tilde{h}}_{j,t+1}^{\left(r\right)}=P_{\text{fwd}}\left(h_{j,t}^{\left(r\right)},c_{j,t}^{\left(r\right)},\Delta \tau_{j,t};\theta_{\text{fwd}}\right). \end{array}$$ (9)

The temporal consistency objective is Equation 10

$$\begin{array}{l} L_{\text{temp}}=\frac{1}{\left|\Omega_{\text{temp}}\right|}\underset{\left(r,j,t\right)\in \Omega_{\text{temp}}}{\sum }{\left\|{\tilde{h}}_{j,t+1}^{\left(r\right)}-h_{j,t+1}^{\left(r\right)}\right\|}_{2}^{2}, \end{array}$$ (10)

where Ω_temp_ contains only valid temporally linked observation pairs. For datasets or samples without such correspondence, no pseudo-successor is constructed and the temporal objective is not evaluated.

For observations with a native spectral axis, suppose that the primary observation is Equation 11

$$\begin{array}{l} x_{j}^{\left(r\right)}={\left[x_{j,0}^{\left(r\right)},\ldots ,x_{j,B-1}^{\left(r\right)}\right]}^{⊤}\in {\mathbb{R}}^{B}, \end{array}$$ (11)

where *B* denotes the number of ordered wavelength bands. For readability, the dataset superscript is omitted in the following spectral definitions. The first *K* discrete frequency components are computed as Equation 12

$$\begin{array}{l} q_{j,\ell }=\frac{1}{B}\overset{B-1}{\underset{b=0}{\sum }}x_{j,b}\exp\left(-\text{i}\frac{2\pi \ell b}{B}\right),\;\;\ell =0,\ldots ,K-1, \end{array}$$ (12)

where *K* is the number of retained low-frequency components. Their magnitudes form the real-valued harmonic descriptor (Equation 13)

$$\begin{array}{l} q_{j}={\left[\left|q_{j,0}|,\ldots ,|q_{j,K-1}\right|\right]}^{⊤}\in {\mathbb{R}}^{K}. \end{array}$$ (13)

The Spectral Consistency Mapper predicts the corresponding descriptor from the current latent state (Equation 14):

$$\begin{array}{l} {\tilde{q}}_{j}=G_{\text{spec}}\left(h_{j};\theta_{\text{spec}}\right)\in {\mathbb{R}}^{K}. \end{array}$$ (14)

The direct spectral-consistency objective is Equation 15

$$\begin{array}{l} L_{\text{spec}}=\frac{1}{\left|\Omega_{\text{spec}}\right|}\underset{j\in \Omega_{\text{spec}}}{\sum }{\left\|{\tilde{q}}_{j}-q_{j}\right\|}_{2}^{2}, \end{array}$$ (15)

where Ω_spec_ contains only observations with native spectral measurements.

To preserve relationships among the retained harmonic components, the normalized harmonic-relation matrix is defined as Equation 16

$$\begin{array}{l} R\left(q\right)=\frac{qq^{⊤}}{{\left\|q\right\|}_{2}^{2}+\epsilon }, \end{array}$$ (16)

where ϵ>0 is a numerical-stability constant. The corresponding harmonic-relation objective is Equation 17

$$\begin{array}{l} L_{\text{harm}}=\frac{1}{\left|\Omega_{\text{spec}}\right|}\underset{j\in \Omega_{\text{spec}}}{\sum }{\left\|R\left({\tilde{q}}_{j}\right)-R\left(q_{j}\right)\right\|}_{F}^{2}. \end{array}$$ (17)

No Fourier-domain descriptor is constructed for image or tabular observations that do not possess a native spectral axis.

Because ${\tilde{q}}_{j}$ lies in the *K*-dimensional harmonic space, it is not directly combined with the *d*-dimensional latent microbial states. Instead, the predicted harmonic descriptor is projected back into the shared latent space (Equation 18):

$$\begin{array}{l} s_{j}^{\left(r\right)}=H_{\text{spec}}\left({\tilde{q}}_{j}^{\left(r\right)};\theta_{\text{map}}\right)\in {\mathbb{R}}^{d}, \end{array}$$ (18)

where $s_{j}^{\left(r\right)}$ denotes latent spectral evidence rather than the raw spectrum.

For any target observation, let $a_{j,\text{temp}}^{\left(r\right)}\in \left\{0,1\right\}$ and $a_{j,\text{spec}}^{\left(r\right)}\in \left\{0,1\right\}$ denote the availability of aligned temporal and spectral evidence, respectively. The Probabilistic Mixture Integrator combines only representations that belong to the shared latent space (Equation 19):

$$\begin{array}{l} r_{j}^{\left(r\right)}=I\left(h_{j}^{\left(r\right)},{\tilde{h}}_{j}^{\left(r\right)},s_{j}^{\left(r\right)};a_{j,\text{temp}}^{\left(r\right)},a_{j,\text{spec}}^{\left(r\right)}\right)\in {\mathbb{R}}^{d}, \end{array}$$ (19)

where unavailable branches are masked before normalization of the evidence weights. Here, ${\tilde{h}}_{j}^{\left(r\right)}$ denotes a forward-projected latent state aligned with observation *j* when a valid temporal predecessor exists.

The integrated state is converted into task probabilities by a dataset-specific prediction head. For a multiclass task (Equation 20),

$$\begin{array}{l} p_{j}^{\left(r\right)}=\mathrm{softmax}\left(W_{o}^{\left(r\right)}r_{j}^{\left(r\right)}+b_{o}^{\left(r\right)}\right)\in {\left[0,1\right]}^{C_{r}}, \end{array}$$ (20)

where Equation 21

$$\begin{array}{l} \overset{C_{r}}{\underset{c=1}{\sum }}p_{j,c}^{\left(r\right)}=1. \end{array}$$ (21)

For a binary prediction task (Equation 22),

$$\begin{array}{l} p_{j}^{\left(r\right)}=\sigma \left(w_{o}^{\left(r\right)⊤}r_{j}^{\left(r\right)}+b_{o}^{\left(r\right)}\right). \end{array}$$ (22)

Thus, the integration mechanism is shared in formulation, whereas the prediction head remains task-specific because the output dimensions and label semantics differ across datasets.

The primary prediction objective is denoted by $L_{\text{task}}$ and corresponds to categorical cross-entropy for multiclass classification or binary cross-entropy for binary classification. The complete objective is Equation 23

$$\begin{array}{l} L=L_{\text{task}}+\lambda_{\text{temp}}L_{\text{temp}}+\lambda_{\text{spec}}L_{\text{spec}}+\lambda_{\text{harm}}L_{\text{harm}}+\lambda_{\text{reg}}{\left\|\Theta \right\|}_{2}^{2}, \end{array}$$ (23)

where Θ denotes all trainable parameters and λ_temp_, λ_spec_, λ_harm_, and λ_reg_ control the corresponding objective terms. Temporal and spectral losses are evaluated only over their valid availability sets. Consequently, the framework can operate on heterogeneous microbial-monitoring datasets without assigning artificial temporal correspondence or spectral structure to observations for which such information is unavailable.

### 3.3 ForwardSpectralProbabilistic

The ForwardSpectralProbabilistic (FSP) model implements the availability-aware formulation introduced in Section 3.2. The framework consists of modality-specific feature extraction, contextual gated fusion, conditional temporal and spectral evidence construction, availability-aware evidence integration, and a task-specific prediction head. The experimental datasets are trained and evaluated independently. Therefore, the term “shared latent space” refers to a common *d*-dimensional representation interface within the FSP formulation rather than to joint training or parameter sharing across datasets. As illustrated in Figure 1, the proposed ForwardSpectralProbabilistic framework first constructs a contextualized current-state representation and then conditionally derives temporal and spectral evidence according to their availability. The available latent representations are subsequently combined by the Probabilistic Mixture Integrator before dataset-specific prediction.

**Figure 1:**
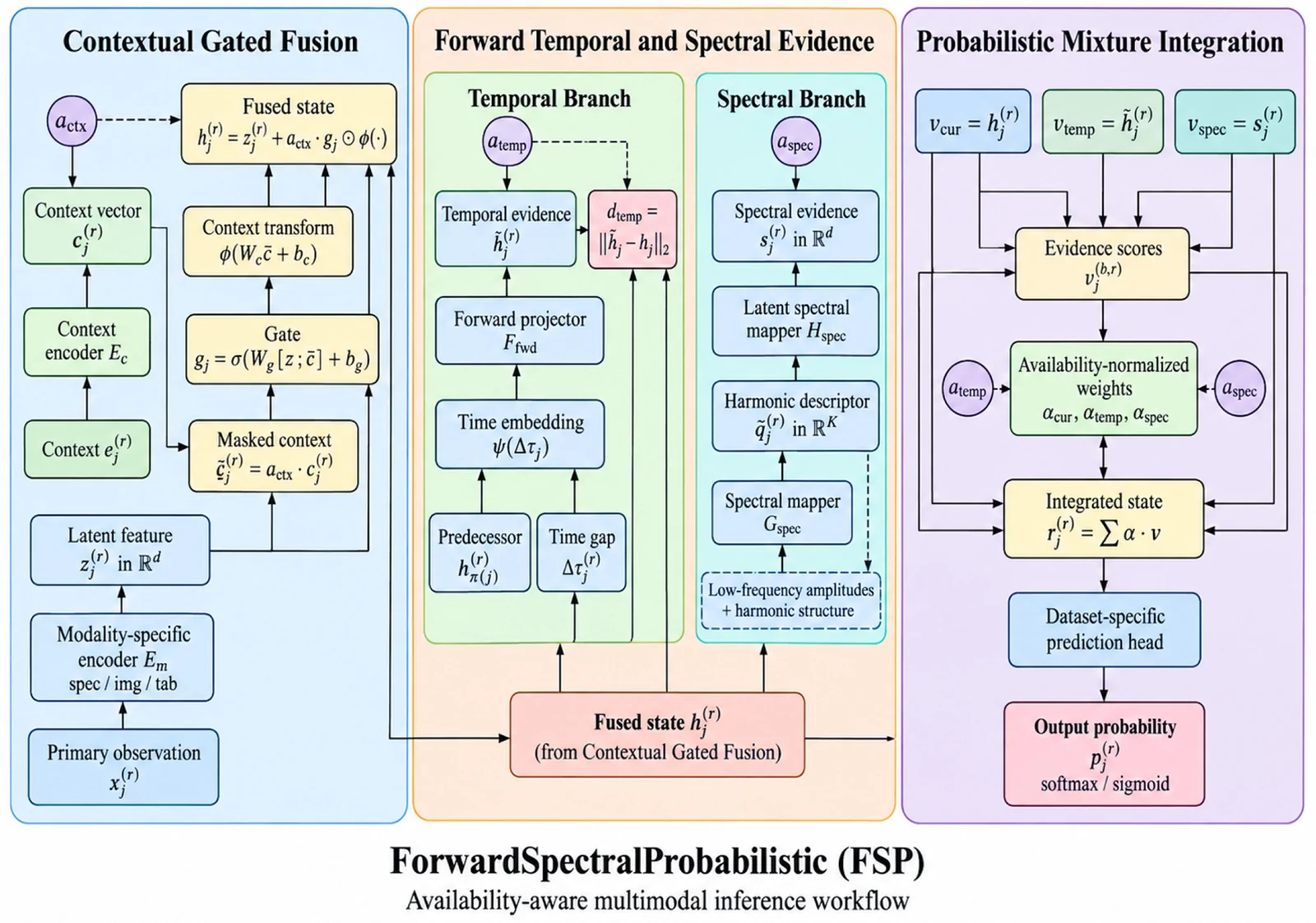
Overview of the ForwardSpectralProbabilistic (FSP) framework. The primary observation is first mapped into a shared latent representation and augmented by available contextual information through contextual gated fusion. Temporal evidence is conditionally generated from a valid predecessor using the Forward Consistency Projector, while spectral evidence is constructed from the predicted harmonic descriptor for observations with a native spectral axis. The current, temporal, and spectral latent representations are then weighted according to their availability and evidence scores by the Probabilistic Mixture Integrator, followed by a dataset-specific prediction head.

Given an observation $x_{j}^{\left(r\right)}$ from dataset *r*, the corresponding modality-specific encoder produces (Equation 24)

$$\begin{array}{l} z_{j}^{\left(r\right)}=E_{m_{r}}\left(x_{j}^{\left(r\right)};\theta_{m_{r}}\right)\in {\mathbb{R}}^{d}, \end{array}$$ (24)

where *m*_*r*_∈{spec, img, tab}. For hyperspectral observations, $E_{\text{spec}}$ operates along the ordered wavelength axis using one-dimensional feature extraction. For microscopy images, $E_{\text{img}}$ extracts two-dimensional spatial and morphological features. For structured observations, $E_{\text{tab}}$ processes standardized numerical variables and encoded categorical variables through fully connected transformations. Although their internal architectures differ, all modality-specific encoders produce representations with the same latent dimensionality *d*.

#### 3.3.1 Contextual gated fusion

When auxiliary environmental, operational, or acquisition variables are available, they are independently encoded as Equation 25

$$\begin{array}{l} c_{j}^{\left(r\right)}=E_{c}\left(e_{j}^{\left(r\right)};\theta_{c}\right)\in {\mathbb{R}}^{d}. \end{array}$$ (25)

Let $a_{j,\text{ctx}}^{\left(r\right)}\in \left\{0,1\right\}$ denote context availability and define the masked contextual representation as Equation 26

$$\begin{array}{l} {\bar{c}}_{j}^{\left(r\right)}=a_{j,\text{ctx}}^{\left(r\right)}c_{j}^{\left(r\right)}. \end{array}$$ (26)

The contextual gate is computed as Equation 27

$$\begin{array}{l} g_{j}^{\left(r\right)}=\sigma \left(W_{g}\left[z_{j}^{\left(r\right)};{\bar{c}}_{j}^{\left(r\right)}\right]+b_{g}\right), \end{array}$$ (27)

and the fused current-state representation is Equation 28

$$\begin{array}{l} h_{j}^{\left(r\right)}=z_{j}^{\left(r\right)}+a_{j,\text{ctx}}^{\left(r\right)}g_{j}^{\left(r\right)}⊙\phi \left(W_{c}{\bar{c}}_{j}^{\left(r\right)}+b_{c}\right), \end{array}$$ (28)

where [·;·] denotes concatenation, σ(·) is the sigmoid function, ϕ(·) denotes a non-linear activation, and ⊙ represents element-wise multiplication. Consequently, $h_{j}^{\left(r\right)}=z_{j}^{\left(r\right)}$ whenever contextual information is unavailable. Context is therefore incorporated before evidence integration and is not treated as an independent branch of the Probabilistic Mixture Integrator.

#### 3.3.2 Forward temporal consistency

Temporal evidence is constructed only for a target observation with a valid temporal predecessor. Let π(*j*) denote the predecessor of observation *j* when such a correspondence exists, and define (Equation 29)

$$\begin{array}{l} \Delta \tau_{j}^{\left(r\right)}=\tau_{j}^{\left(r\right)}-\tau_{\pi \left(j\right)}^{\left(r\right)}. \end{array}$$ (29)

The Forward Consistency Projector generates a latent state aligned with the target observation *j* (Equation 30):

$$\begin{array}{l} {\tilde{h}}_{j}^{\left(r\right)}=h_{\pi \left(j\right)}^{\left(r\right)}+\Delta \tau_{j}^{\left(r\right)}F_{\text{fwd}}\left(\left[h_{\pi \left(j\right)}^{\left(r\right)};{\bar{c}}_{\pi \left(j\right)}^{\left(r\right)};\psi \left(\Delta \tau_{j}^{\left(r\right)}\right)\right];\theta_{\text{fwd}}\right), \end{array}$$ (30)

where ψ(·) denotes an elapsed-time embedding and $F_{\text{fwd}}$ is a residual transition network. The projector therefore estimates the latent change associated with the interval between a valid predecessor and the current target rather than reconstructing the raw future observation.

For a valid temporal pair, the projection discrepancy is Equation 31

$$\begin{array}{l} d_{j}^{\text{temp}}=\|{\tilde{h}}_{j}^{\left(r\right)}-h_{j}^{\left(r\right)}|{|}_{2}. \end{array}$$ (31)

Its squared form contributes to $L_{\text{temp}}$ defined in Section 3.2. During inference, ${\tilde{h}}_{j}^{\left(r\right)}$ can serve as additional evidence for observation *j* only when its valid predecessor is available. Otherwise, $a_{j,\text{temp}}^{\left(r\right)}=0$ and the temporal branch is excluded. Thus, the forward branch provides temporally aligned auxiliary evidence and is not interpreted as a standalone long-horizon forecasting model.

#### 3.3.3 Spectral consistency and harmonic preservation

For observations with a native spectral axis, the Spectral Consistency Mapper predicts the harmonic descriptor defined in Section 3.2 (Equation 32):

$$\begin{array}{l} {\tilde{q}}_{j}^{\left(r\right)}=G_{\text{spec}}\left(h_{j}^{\left(r\right)};\theta_{\text{spec}}\right)\in {\mathbb{R}}^{K}. \end{array}$$ (32)

The mapper is implemented as a non-linear projection from the common latent space to the *K* retained harmonic amplitudes. Its parameters are supervised through both $L_{\text{spec}}$ and $L_{\text{harm}}$, encouraging the current latent representation to retain information associated with individual low-frequency amplitudes as well as their relative harmonic structure.

Because ${\tilde{q}}_{j}^{\left(r\right)}\in {\mathbb{R}}^{K}$ does not belong to the same representation space as $h_{j}^{\left(r\right)}\in {\mathbb{R}}^{d}$, it is mapped back into the shared latent dimensionality before evidence integration (Equation 33):

$$\begin{array}{l} s_{j}^{\left(r\right)}=H_{\text{spec}}\left({\tilde{q}}_{j}^{\left(r\right)};\theta_{\text{map}}\right)=\phi \left(W_{s}{\tilde{q}}_{j}^{\left(r\right)}+b_{s}\right)\in {\mathbb{R}}^{d}. \end{array}$$ (33)

Here, $s_{j}^{\left(r\right)}$ denotes latent spectral evidence rather than the raw input spectrum. For observations without a native spectral axis, $a_{j,\text{spec}}^{\left(r\right)}=0$ and no spectral or Fourier-domain evidence is constructed.

#### 3.3.4 Probabilistic mixture integration

The Probabilistic Mixture Integrator combines only evidence represented in the common *d*-dimensional latent space. For observation *j*, the candidate representations are Equation 34

$$\begin{array}{l} v_{j}^{\left(\text{cur},r\right)}=h_{j}^{\left(r\right)},\;\;v_{j}^{\left(\text{temp},r\right)}={\tilde{h}}_{j}^{\left(r\right)},\;\;v_{j}^{\left(\text{spec},r\right)}=s_{j}^{\left(r\right)}. \end{array}$$ (34)

Their availability indicators are Equation 35

$$\begin{array}{l} a_{j,\text{cur}}^{\left(r\right)}=1,\;\;a_{j,\text{temp}}^{\left(r\right)}\in \left\{0,1\right\},\;\;a_{j,\text{spec}}^{\left(r\right)}\in \left\{0,1\right\}. \end{array}$$ (35)

For each available branch *b*∈{cur, temp, spec}, an evidence score is computed as Equation 36

$$\begin{array}{l} u_{j}^{\left(b,r\right)}=w_{b}^{⊤}\tanh\left(W_{b}v_{j}^{\left(b,r\right)}+b_{b}\right). \end{array}$$ (36)

The scores are normalized only over available branches (Equation 37):

$$\begin{array}{l} \alpha_{j}^{\left(b,r\right)}=\frac{a_{j,b}^{\left(r\right)}\exp\left(u_{j}^{\left(b,r\right)}\right)}{\underset{b^{\prime }}{\sum }a_{j,b^{\prime }}^{\left(r\right)}\exp\left(u_{j}^{\left(b^{\prime },r\right)}\right)}. \end{array}$$ (37)

Because $a_{j,\text{cur}}^{\left(r\right)}=1$, the denominator remains well defined even when no auxiliary branch is available. The resulting integrated state is Equation 38

$$\begin{array}{l} r_{j}^{\left(r\right)}=\underset{b}{\sum }\alpha_{j}^{\left(b,r\right)}v_{j}^{\left(b,r\right)}\in {\mathbb{R}}^{d}. \end{array}$$ (38)

If neither temporal nor spectral evidence is available, the formulation reduces naturally to Equation 39

$$\begin{array}{l} r_{j}^{\left(r\right)}=h_{j}^{\left(r\right)}. \end{array}$$ (39)

The coefficients $\alpha_{j}^{\left(b,r\right)}$ are therefore availability-normalized mixture weights on the probability simplex. They provide adaptive evidence weighting but are not intended to represent a Bayesian posterior over model parameters.

The Probabilistic Mixture Integrator itself produces the integrated latent representation $r_{j}^{\left(r\right)}$. The final predictive probability is generated by the dataset-specific task head. For multiclass classification (Equation 40),

$$\begin{array}{l} p_{j}^{\left(r\right)}=\mathrm{softmax}\left(W_{o}^{\left(r\right)}r_{j}^{\left(r\right)}+b_{o}^{\left(r\right)}\right), \end{array}$$ (40)

where $p_{j}^{\left(r\right)}\in {\left[0,1\right]}^{C_{r}}$ and Equation 41

$$\begin{array}{l} \overset{C_{r}}{\underset{c=1}{\sum }}p_{j,c}^{\left(r\right)}=1. \end{array}$$ (41)

For binary pathogen-risk or microbial-marker prediction (Equation 42),

$$\begin{array}{l} p_{j}^{\left(r\right)}=\sigma \left(w_{o}^{\left(r\right)⊤}r_{j}^{\left(r\right)}+b_{o}^{\left(r\right)}\right). \end{array}$$ (42)

The prediction heads are dataset-specific because the number and semantics of the target classes differ, whereas the conditional evidence-integration formulation remains unchanged.

All model parameters within a dataset-specific experiment are optimized jointly using the complete objective defined in Section 3.2. Temporal, spectral-consistency, and harmonic-relation losses are evaluated only over their corresponding valid availability sets. Consequently, unavailable evidence does not contribute an artificial zero-valued target or auxiliary penalty.

At inference time, FSP proceeds in four stages. The primary observation is encoded using the modality-specific encoder. Available contextual information is incorporated through gated residual fusion. Aligned temporal evidence and latent spectral evidence are constructed only when their required information is available. The Probabilistic Mixture Integrator combines the available latent representations, and the dataset-specific prediction head produces the class or pathogen-risk probability. This conditional computation mechanism preserves the native information structure of each sensing modality while maintaining a consistent prediction formulation across heterogeneous microbial-monitoring tasks.

### 3.4 Harmonic state projection

Harmonic State Projection (HSP) is formulated as a structured regularization strategy within the ForwardSpectralProbabilistic framework rather than as an independent prediction module. Its purpose is to impose auxiliary constraints on the shared latent representation when temporal or spectral structure is actually supported by the observations. HSP contains two complementary components: a temporal projection constraint for observations with valid predecessor-target correspondence and a harmonic relation constraint for observations with a native spectral axis. These constraints regularize different aspects of the latent representation and are activated independently according to data availability. Figure 2 summarizes the Harmonic State Projection strategy. HSP imposes temporal and spectral regularization only on observations for which the corresponding structural information is available, while the resulting auxiliary losses are incorporated into the complete training objective together with the primary task loss and parameter regularization.

**Figure 2:**
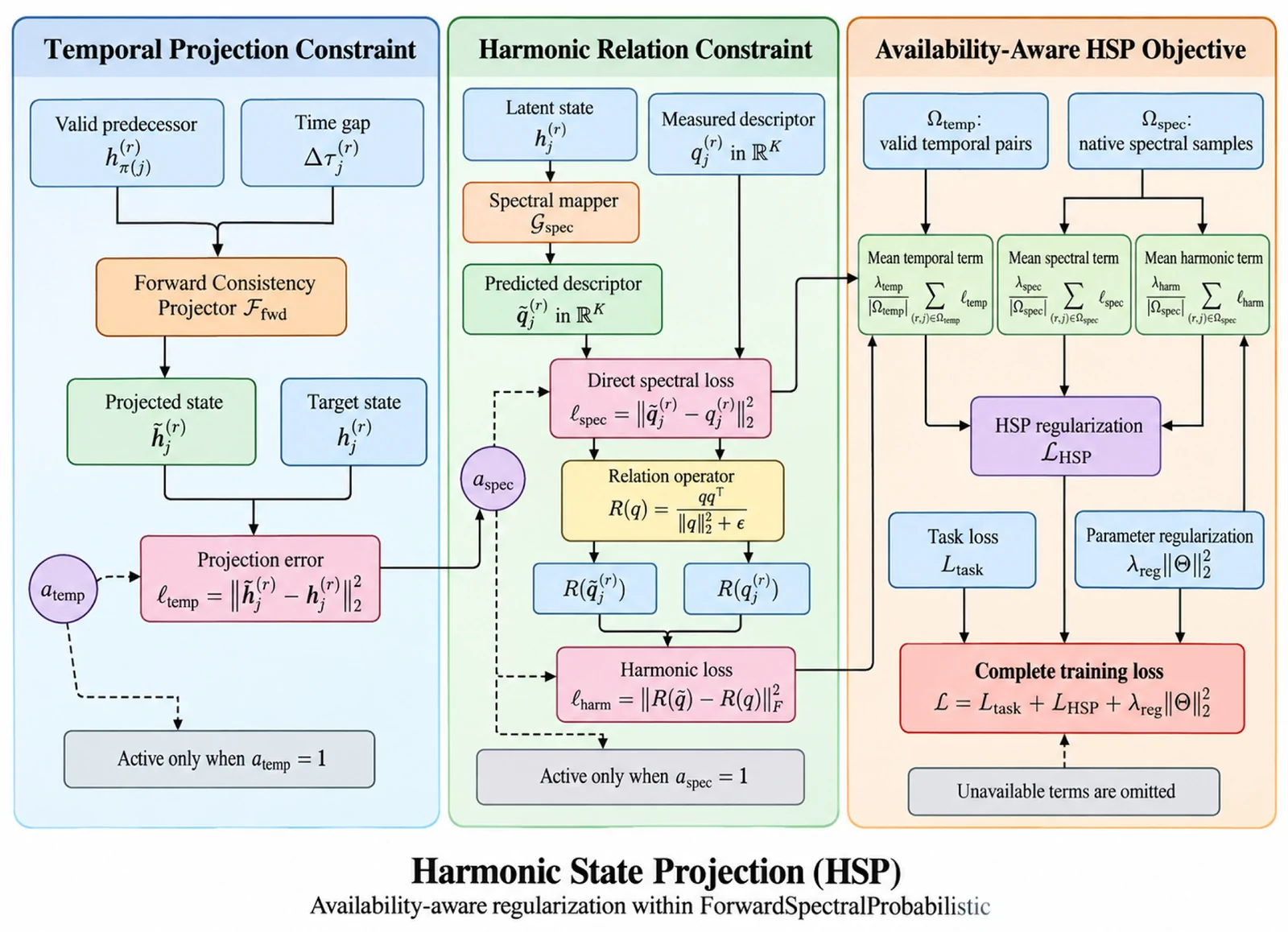
Schematic illustration of the Harmonic State Projection (HSP) strategy. The temporal projection constraint penalizes disagreement between the predecessor-based projected latent state and the observed target latent state. The harmonic relation constraint jointly preserves the amplitudes of the retained spectral components and their normalized pairwise relationships. Temporal, spectral, and harmonic penalties are evaluated only over their corresponding availability sets and are combined into the availability-aware HSP regularization objective. The complete training loss further includes the primary task objective and parameter regularization.

#### 3.4.1 Temporal projection constraint

As defined in Section 3.3, let π(*j*) denote a valid temporal predecessor of target observation *j*. The Forward Consistency Projector produces a latent representation ${\tilde{h}}_{j}^{\left(r\right)}$ aligned with the target state $h_{j}^{\left(r\right)}$. For a valid temporal pair, the sample-level projection error is defined as Equation 43

$$\begin{array}{l} \ell_{j,\text{temp}}^{\left(r\right)}={\left\|{\tilde{h}}_{j}^{\left(r\right)}-h_{j}^{\left(r\right)}\right\|}_{2}^{2}. \end{array}$$ (43)

This constraint operates entirely in the shared latent space and therefore does not require reconstruction of the original spectral, image, or tabular observation. A smaller projection error indicates stronger agreement between the state predicted from the temporal predecessor and the representation of the subsequently observed target. Importantly, the discrepancy is interpreted as a training-consistency measure rather than as direct evidence of abnormal microbial behavior.

The temporal constraint is evaluated only when $a_{j,\text{temp}}^{\left(r\right)}=1$. Observations without a defensible temporal predecessor are excluded from this objective, and no artificial temporal ordering is introduced.

#### 3.4.2 Harmonic relation constraint

For an observation with a native spectral axis, Section 3.2 defines the measured harmonic descriptor $q_{j}^{\left(r\right)}\in {\mathbb{R}}^{K}$ and the corresponding descriptor predicted from the latent state, ${\tilde{q}}_{j}^{\left(r\right)}\in {\mathbb{R}}^{K}$. Direct spectral agreement is measured by Equation 44

$$\begin{array}{l} \ell_{j,\text{spec}}^{\left(r\right)}={\left\|{\tilde{q}}_{j}^{\left(r\right)}-q_{j}^{\left(r\right)}\right\|}_{2}^{2}. \end{array}$$ (44)

HSP additionally constrains the relationships among the retained harmonic components. Using the normalized relation operator introduced in Section 3.2 (Equation 45),

$$\begin{array}{l} R\left(q\right)=\frac{qq^{⊤}}{{\left\|q\right\|}_{2}^{2}+\epsilon }, \end{array}$$ (45)

the sample-level harmonic discrepancy is defined as Equation 46

$$\begin{array}{l} \ell_{j,\text{harm}}^{\left(r\right)}={\left\|R\left({\tilde{q}}_{j}^{\left(r\right)}\right)-R\left(q_{j}^{\left(r\right)}\right)\right\|}_{F}^{2}. \end{array}$$ (46)

The direct spectral term preserves the amplitudes of the retained harmonic components, whereas the relation term preserves their normalized pairwise structure. These two constraints are therefore complementary: the former penalizes coefficient-level disagreement, while the latter regularizes the relative organization of the low-frequency spectral representation.

Both spectral constraints are evaluated only when $a_{j,\text{spec}}^{\left(r\right)}=1$. Consequently, no Fourier-domain target, harmonic descriptor, or spectral penalty is constructed for microscopy images or structured observations that do not possess a native wavelength axis.

#### 3.4.3 Availability-aware HSP objective

Let Ω_temp_ denote the set of observations with valid temporal correspondence and Ω_spec_ denote the set of observations containing native spectral measurements. The HSP regularization objective is defined as Equation 47

$$\begin{array}{l} \begin{array}{cc} L_{\text{HSP}}= & \frac{\lambda_{\text{temp}}}{\left|\Omega_{\text{temp}}\right|}\underset{\left(r,j\right)\in \Omega_{\text{temp}}}{\sum }\ell_{j,\text{temp}}^{\left(r\right)}\\ & +\frac{\lambda_{\text{spec}}}{\left|\Omega_{\text{spec}}\right|}\underset{\left(r,j\right)\in \Omega_{\text{spec}}}{\sum }\ell_{j,\text{spec}}^{\left(r\right)}\\ & +\frac{\lambda_{\text{harm}}}{\left|\Omega_{\text{spec}}\right|}\underset{\left(r,j\right)\in \Omega_{\text{spec}}}{\sum }\ell_{j,\text{harm}}^{\left(r\right)}. \end{array} \end{array}$$ (47)

A term whose corresponding availability set is empty is omitted from the objective. The complete training criterion can therefore be written as Equation 48

$$\begin{array}{l} L=L_{\text{task}}+L_{\text{HSP}}+\lambda_{\text{reg}}{\left\|\Theta \right\|}_{2}^{2}, \end{array}$$ (48)

which is directly equivalent to the availability-aware objective defined in Section 3.2.

The temporal and spectral components of HSP should not be interpreted as requiring both information sources for the same observation. A temporally linked non-spectral observation can receive temporal supervision without any spectral constraint, whereas a hyperspectral observation can receive spectral and harmonic supervision without requiring a temporal predecessor. Observations containing neither form of auxiliary information are optimized through the primary task objective and parameter regularization.

HSP also remains distinct from the Probabilistic Mixture Integrator. The HSP terms regulate how temporal and spectral information is represented during training, whereas the integrator described in Section 3.3 determines how available latent evidence contributes to the prediction of an individual observation. The temporal branch provides ${\tilde{h}}_{j}^{\left(r\right)}\in {\mathbb{R}}^{d}$, while the spectral branch transforms ${\tilde{q}}_{j}^{\left(r\right)}$ into latent spectral evidence $s_{j}^{\left(r\right)}\in {\mathbb{R}}^{d}$. The integrator then combines $h_{j}^{\left(r\right)}$, ${\tilde{h}}_{j}^{\left(r\right)}$, and $s_{j}^{\left(r\right)}$ only when the corresponding evidence is available. Thus, HSP regularization and probabilistic evidence integration play complementary but non-redundant roles within the FSP framework.

## 4 Experimental setup

### 4.1 Dataset

Four publicly accessible microbial-safety datasets were used to evaluate the proposed framework under heterogeneous sensing and monitoring conditions. These datasets cover hyperspectral microbial signatures, microscopy images, structured pathogen-surveillance records, and agricultural-water measurements. Because their raw modalities and prediction targets differ, the datasets are trained and evaluated independently and are not merged at the sample level. Each dataset retains its native representation, while the corresponding variables are assigned to primary observations, contextual information, and auxiliary temporal or spectral evidence according to the availability-aware formulation introduced in Section 3.2. Table 1 summarizes the experimental roles of the four datasets.

**Table 1:** Summary of the datasets and their roles in the availability-aware FSP framework.

Dataset	Prediction task	Primary observation	Context	Temporal	Spectral	Evaluation protocol
Salmonella serovar hyperspectral	5-class serovar classification	303-band single-cell spectrum	—	—	✓	5-fold grouped evaluation by source hypercube
Microcolony domain adaptation	6-class bacterial classification	Microscopy image	Acquisition metadata	—	—	539 training/90 standard test/420 OOD test; 150 few-shot images excluded from the main experiment
USDA raw poultry	Salmonella and campylobacter presence	Non-outcome sampling and geographic variables	Current and lagged meteorological variables	✓^*^	—	5-fold grouped evaluation by establishment
PAWQ agricultural water	Four microbial-source-marker tasks	Water-quality and non-target microbiological variables	Weather, land cover, livestock proximity, and infrastructure	—	—	Stratified 5-fold cross-validation

A check mark indicates that the corresponding information source is used by the proposed model, whereas “—” indicates that the branch is disabled. ^*^Temporal evidence is used only for records with a valid chronological predecessor from the same establishment; other records remain in the primary classification task but are excluded from the temporal-consistency objective.

The Salmonella Serovar Hyperspectral Dataset (Papa et al., 2025) contains hyperspectral measurements acquired for classification of five foodborne *Salmonella* serovars: *S. Enteritidis, S. 4,[5],12:i:-, S. Infantis, S. Johannesburg*, and *S. Kentucky*. The original acquisition comprises 500 hyperspectral data cubes, with 100 cubes for each serovar. The wavelength range is approximately 399-1000 nm and is represented by 303 spectral bands. The released single-cell representation contains 25,972 segmented spectra whose reflectance values are provided after Standard Normal Variate (SNV) normalization. The data and associated code are available through Zenodo (https://doi.org/10.5281/zenodo.16740800). For this dataset, the 303-band single-cell spectrum constitutes the primary observation $x_{j}^{\left(r\right)}$. No separate environmental or acquisition context is used in the main experiment, so the contextual contribution is disabled. Because the input has a native ordered wavelength axis, the spectral availability indicator satisfies $a_{j,\text{spec}}^{\left(r\right)}=1$, and the Spectral Consistency Mapper and harmonic-relation constraint are applicable. No temporal correspondence is assumed, and therefore $a_{j,\text{temp}}^{\left(r\right)}=0$. Spectra originating from the same hyperspectral cube are retained in the same evaluation fold to reduce acquisition-level information leakage. The Microcolony Domain Adaptation Dataset (Bhattacharya et al., 2025) contains microscopy images from six food-related bacterial classes: *Bacillus coagulans, Bacillus subtilis, Escherichia coli* 1612, *Listeria innocua, Salmonella Enteritidis*, and *Salmonella Typhimurium*. The dataset contains 539 standard training images, 150 labeled few-shot target images, 90 standard-domain test images, and 420 out-of-distribution (OOD) test images, giving 1,199 images in total. The domain variation includes changes in magnification, incubation duration, imaging modality, focus, and agar concentration. The complete dataset is available at https://doi.org/10.5281/zenodo.16741157. For the proposed model, the microscopy image is treated as the primary observation. Acquisition descriptors available for the corresponding image, including microscopy modality, magnification, incubation duration, focus, and agar-related acquisition information, are treated as contextual variables when provided. These variables are encoded separately by the contextual encoder and are not concatenated with the raw image. The dataset does not provide a native spectral axis for the image observations and is not modeled as a longitudinal sequence; consequently, the spectral and temporal auxiliary branches are disabled. The predefined standard-domain and OOD test partitions are retained. The 150-image few-shot subset is excluded from the source-only experiments reported in this study.

The USDA Raw Poultry Salmonella Campylobacter Dataset is derived from the U.S. Department of Agriculture Food Safety and Inspection Service (USDA FSIS) Raw Poultry Sampling Program (U.S. Department of Agriculture, Food Safety and Inspection Service). The experimental snapshot contains 4,887 raw chicken carcass samples from FSIS-regulated establishments across 36 U.S. states, with 71 recorded fields. The records contain pathogen-testing outcomes together with sampling, geographic, temporal, and meteorological information. Meteorological measurements include temperature, precipitation, humidity, pressure, wind, cloud cover, and related variables for the sampling day and the preceding 3 days. SalmonellaSPAnalysis and CampylobacterAnalysis30ml are treated as two independent binary prediction targets. In the FSP formulation, non-outcome sampling and geographic attributes form the primary structured observation, whereas current and lagged meteorological variables are treated as contextual information. Variables that directly reveal the pathogen-testing outcome are excluded from the predictor set. The establishment identifier is used only as the grouping variable for evaluation and is not supplied to the predictive model. Sampling time is retained for construction of temporal correspondence. A temporal predecessor is considered valid only when a chronologically earlier record from the same establishment can be linked to the target record; only such observations satisfy $a_{j,\text{temp}}^{\left(r\right)}=1$. Records without a valid predecessor remain in the primary classification task but do not contribute to the temporal consistency objective. Because the dataset has no native spectral axis, the spectral branch is disabled. The official source is the USDA FSIS Laboratory Sampling Data repository (https://www.fsis.usda.gov/science-data/data-sets-visualizations/laboratory-sampling-data), and the standardized experimental snapshot is available at https://huggingface.co/datasets/food-ai-nexus/chicken-salmonella-campylobacter-us-facilities. The Predicting Agricultural Water Quality (PAWQ) Dataset (Green et al., 2021; Weller et al., 2020) contains 196 observations from 68 non-overlapping watersheds in Upstate New York and comprises 95 variables describing water quality, weather, land cover, livestock proximity, and infrastructure. Microbial measurements include *E. coli* concentration and four presence/absence microbial-source-tracking markers: HF183_pa, Rum2Bac_pa, DG3_pa, and GFD_pa. The four marker-presence variables are evaluated as separate binary prediction targets, and dataset-level results are obtained by averaging the target-specific metrics with equal weight. The original data and documentation are available at https://doi.org/10.5281/zenodo.18500867. For this dataset, water-quality and non-target microbiological measurements are treated as the primary structured observation, while weather, land-cover, livestock-proximity, and infrastructure variables are represented as contextual information. The variable corresponding directly to the prediction target is removed from the predictor set for each task. Because the records do not provide a sample-level temporal predecessor target relation suitable for the forward projection mechanism, the temporal auxiliary branch is disabled. The observations also contain no native wavelength axis, so the spectral branch is not applied. Stratified five-fold cross-validation is used for each binary target because of the limited sample size and class distribution. The four datasets provide different combinations of information rather than a common multimodal observation. The FSP framework therefore does not require each sample to contain image, spectral, temporal, and contextual data simultaneously. Dataset-specific preprocessing is applied first, after which only the evidence supported by the corresponding dataset is activated. Detailed preprocessing, model implementation, data partitioning, and training settings are provided in Section 4.2.

### 4.2 Experimental details

All experiments were implemented in PyTorch and executed on NVIDIA Tesla V100 GPUs. Because the four datasets contain different input modalities and different combinations of auxiliary information, preprocessing was performed separately for primary observations and contextual variables according to the roles defined in Section 4.1. All normalization, imputation, categorical encoding, dimensionality transformation, and other data-dependent preprocessing parameters were estimated exclusively from the corresponding training partition and were subsequently applied to validation and test data. No statistics computed from validation or test observations were used to fit the preprocessing transformations.

#### 4.2.1 Data preprocessing

For the Salmonella Serovar Hyperspectral Dataset, the released single-cell spectra were processed in their native wavelength order. The provided Standard Normal Variate (SNV)-normalized 303-band representation was used directly as the primary input, and no additional image-based augmentation, cropping, flipping, or color transformation was applied. The harmonic descriptor used by the spectral auxiliary branch was computed from the same SNV-normalized spectrum. Spectra originating from the same hyperspectral cube were assigned to the same partition throughout evaluation to prevent cells from a common acquisition from appearing in both training and evaluation folds. No independent contextual variables or temporal pairs were constructed for this dataset. For the Microcolony Domain Adaptation Dataset, microscopy images were resized to 224 × 224 pixels. Training-only augmentation consisted of random resized cropping with a scale range of [0.90, 1.00], random horizontal and vertical flipping with probability 0.5, and random rotation within ±10°. Color jittering was not applied because artificial modification of microscopy intensity and color characteristics could alter biologically relevant visual information. The predefined standard-domain and out-of-distribution (OOD) test images were not augmented.

Acquisition descriptors associated with each microscopy image were processed separately from the image representation and used as contextual information. Continuous acquisition variables were standardized using statistics estimated from the training partition, whereas categorical descriptors were encoded using training-derived one-hot representations with an additional category for previously unseen values. The same contextual variables were used by the context-aware comparison model described in Section 4.3 so that any performance difference could not be attributed to access to additional metadata. The 150 labeled few-shot target images were excluded from the source-only experiments reported in this study and were not used for parameter estimation, hyperparameter selection, early stopping, or evaluation of the main comparison models. No temporal or spectral auxiliary branch was activated for the Microcolony experiments. For the USDA Raw Poultry Dataset, predictors were divided into the primary and contextual groups defined in Section 4.1. Non-outcome sampling and geographic attributes constituted the primary structured input, whereas current and lagged meteorological measurements were processed through the contextual encoder. Numerical variables in both groups were median-imputed and standardized using statistics estimated from the training fold. Categorical variables were transformed using training-derived one-hot encoding, with an additional category for unseen values. Establishment identifiers were used only to construct grouped data partitions and temporal correspondences and were excluded from the predictive feature set. Variables directly revealing either pathogen-testing outcome were also removed. Temporal correspondence was constructed independently within each training or evaluation partition after grouping constraints had been applied. For a target observation *j*, its temporal predecessor π(*j*) was defined as the chronologically closest record from the same establishment with a strictly earlier sampling time. The elapsed interval Δτ_*j*_ = τ_*j*_−τ_π(*j*)_ was expressed in days. A record was assigned *a*_*j*, temp_ = 1 only when such a predecessor existed; otherwise, *a*_*j*, temp_ = 0. Records without a valid predecessor remained in the primary pathogen-classification task but did not contribute to $L_{\text{temp}}$. Temporal links were never constructed across establishments or across data partitions. For the PAWQ Agricultural Water Dataset, water-quality and non-target microbiological variables were processed as the primary structured input, whereas weather, land-cover, livestock-proximity, and infrastructure variables were processed as contextual information. Numerical variables were median-imputed and standardized using training-fold statistics. Categorical variables, when present, were encoded from the training partition only. For each microbial-source-marker task, the variable corresponding directly to the prediction target was removed before model fitting. Because the PAWQ records do not provide a defensible sample-level predecessor target relationship or a native wavelength axis, the temporal and spectral auxiliary branches were disabled. For the structured-data comparisons on USDA and PAWQ, all tabular baselines received the union of the primary and contextual predictor variables used by the proposed framework. The proposed method therefore did not receive additional predictive covariates; its distinction lies in representing primary and contextual variables through separate encoders before gated fusion.

#### 4.2.2 Model implementation

The common latent dimensionality was set to *d* = 128. The hyperspectral encoder consisted of three one-dimensional convolutional blocks with 32, 64, and 128 output channels and kernel sizes of 7, 5, and 3, respectively. Each convolutional block was followed by batch normalization and GELU activation, and adaptive average pooling was used to obtain the final 128-dimensional representation. The microscopy-image encoder used a ResNet-18 backbone initialized with ImageNet-pretrained weights. Its original classification layer was removed and replaced by a linear projection to the 128-dimensional latent space. The structured-data encoder consisted of three fully connected layers with hidden dimensions of 256, 128, and 128, respectively. GELU activation was used between layers and dropout was applied with probability 0.2. Contextual variables were processed by a two-layer multilayer perceptron whose output dimensionality was 128. The contextual representation was combined with the primary latent representation using the gated residual fusion operator defined in Section 3.3. When no contextual information was available, the corresponding availability mask forced the fused representation to reduce to the primary representation. For temporal modeling, elapsed time was represented by a 16-dimensional embedding before being concatenated with the predecessor latent state and its contextual representation. The Forward Consistency Projector was implemented as a two-layer residual multilayer perceptron with a 128-dimensional hidden representation and GELU activation. Its output represented the latent state-change vector used to construct the temporally aligned state described in Section 3.3. For the Spectral Consistency Mapper, the number of retained harmonic components was set to *K* = 16. The mapper used a 128 → 64 → *K* non-linear projection with GELU activation. The predicted harmonic descriptor was subsequently transformed into latent spectral evidence using a *K* → 64 → 128 projection before mixture integration. The evidence-scoring network of the Probabilistic Mixture Integrator used a hidden dimensionality of 64. Availability-normalized evidence weights were computed only over the current, temporal, and spectral representations present for the corresponding observation. The final prediction layer was a dataset-specific linear head with either softmax output for multiclass tasks or sigmoid output for binary tasks.

#### 4.2.3 Training protocol

All newly initialized parameters were optimized using Adam with an initial learning rate of 1 × 10^−3^. The ImageNet-pretrained ResNet-18 backbone was fine-tuned using a smaller initial learning rate of 1 × 10^−4^. The learning rates were updated using cosine annealing to a minimum value of 1 × 10^−6^. The regularization coefficient was set to $\lambda_{\text{reg}}=5\times 10^{-4}$ and was implemented through the optimizer weight-decay term rather than by adding a duplicate penalty during optimization. The auxiliary-objective coefficients were fixed at Equation 49

$$\begin{array}{l} \lambda_{\text{temp}}=0.20,\;\;\lambda_{\text{spec}}=0.10,\;\;\lambda_{\text{harm}}=0.10. \end{array}$$ (49)

These auxiliary terms were evaluated only over the availability sets defined in Sections 3.2 and 3.4. The reported architectural and loss-weight settings were selected using training and validation data and were fixed before evaluation on the corresponding test partitions. Batch sizes of 64 were used for the hyperspectral and structured-data experiments, whereas a batch size of 32 was used for microscopy images. Training was performed for a maximum of 150 epochs. The checkpoint achieving the highest validation F1-score was retained, and early stopping was triggered when validation F1 did not improve for 20 consecutive epochs. Macro-F1 was used for model selection in multiclass tasks, whereas positive-class F1 was used for binary tasks. For imbalanced classification tasks, class weights were derived exclusively from the class frequencies of the training partition and normalized to have mean weight one. These weights were incorporated into the corresponding categorical or binary cross-entropy objective. The test partitions were never used to determine class weights, architectural settings, loss coefficients, learning rates, or stopping epochs.

#### 4.2.4 Data partitioning and repeated experiments

The Salmonella hyperspectral dataset was evaluated using five-fold grouped cross-validation, with the source hyperspectral cube serving as the grouping unit. All spectra from one cube therefore remained in the same fold. Within each outer training partition, a further grouped split reserved 10% of the available training groups for validation. The USDA dataset was evaluated using five-fold grouped cross-validation by establishment. The same establishment grouping was applied to both pathogen prediction targets, and 10% of the establishments in each outer training partition were reserved for validation. Temporal predecessor target pairs were constructed only after these partitions had been created, preventing temporal links from crossing training, validation, or test boundaries. The PAWQ dataset was evaluated separately for each of the four binary marker tasks using stratified five-fold cross-validation. Within each outer training fold, a stratified 10% subset was reserved for validation. Because the class labels differ across marker tasks, the stratified folds were generated separately for each target. For the Microcolony dataset, the predefined source-training, standard-domain-test, and OOD-test partitions were retained. A stratified 10% subset of the 539 source-training images was reserved for validation. The 150 labeled few-shot target images were not incorporated into any source-only training or validation partition. Each experimental configuration was repeated using five independent random seeds: 13, 31, 47, 73, and 101. The seed controlled model initialization, training-data order, stochastic augmentation, and generation of repeated cross-validation or validation partitions where applicable. Identical partitions and seeds were used for the proposed method and the corresponding comparison methods. For the cross-validation experiments, out-of-fold predictions were first collected across the five test folds for each seed. Dataset-level metrics were then calculated from these out-of-fold predictions and summarized across the five seeds. For the fixed Microcolony test sets, each trained seed produced one set of standard-domain and OOD predictions. Unless otherwise stated, Tables 2–4 report the mean and standard deviation across the five repeated experiments.

Table 2Comparison with modality-matched methods on the Salmonella hyperspectral and Microcolony microscopy datasets.

**Table:** 

(a) Salmonella Serovar Hyperspectral Dataset				
Model	Accuracy	Recall	F1 Score	AUC
PCA-kNN (Papa et al., 2025)	73.62 ± 0.91	72.84 ± 1.13	72.11 ± 1.06	88.37 ± 0.78
PCA-SVM (Papa et al., 2025)	76.48 ± 0.74	75.66 ± 0.92	75.28 ± 0.85	90.41 ± 0.61
PCA-RF (Papa et al., 2025)	78.21 ± 0.68	77.55 ± 0.81	76.93 ± 0.87	91.16 ± 0.57
PCA-MLP (Papa et al., 2025)	80.73 ± 0.63	79.94 ± 0.79	79.61 ± 0.72	92.53 ± 0.49
LSTM-HMI (Kang et al., 2021)	81.94 ± 0.71	81.22 ± 0.77	80.88 ± 0.73	93.47 ± 0.51
1D-CNN (Kang et al., 2020)	82.66 ± 0.54	81.87 ± 0.70	81.59 ± 0.64	94.08 ± 0.43
**FSP**	**84.91 ± 0.47**	**84.26 ± 0.58**	**83.97 ± 0.55**	**95.31 ± 0.36**

**Table:** 

(b) Microcolony Domain Adaptation Dataset						
Model	Standard-domain test			OOD test		
	Accuracy	F1 Score	AUC	Accuracy	F1 Score	AUC
ResNet-18 (He et al., 2016)	92.84 ± 0.88	92.41 ± 0.95	97.62 ± 0.44	54.78 ± 1.82	52.96 ± 2.10	75.84 ± 1.22
EfficientNetV2-S (Tan and Le, 2021)	**95.74 ± 0.64**	95.18 ± 0.69	98.79 ± 0.27	62.37 ± 1.47	60.88 ± 1.69	81.26 ± 0.94
ConvNeXt-Tiny (Liu et al., 2022)	94.12 ± 0.79	93.76 ± 0.84	98.16 ± 0.37	59.63 ± 1.61	58.24 ± 1.74	79.45 ± 1.03
ResNet-18+Context	95.11 ± 0.66	94.75 ± 0.71	98.58 ± 0.29	65.21 ± 1.36	63.74 ± 1.52	82.73 ± 0.81
**FSP**	95.61 ± 0.58	**95.34 ± 0.61**	**98.87 ± 0.24**	**67.43 ± 1.29**	**65.92 ± 1.44**	**84.11 ± 0.76**

Values are percentages and are reported as mean ± standard deviation over five independent random seeds. Recall, F1, and AUC are macro-averaged for multiclass tasks. Boldface indicates the highest mean value in each column and does not by itself denote statistical significance.

#### 4.2.5 Evaluation metrics

The primary reported metrics were accuracy, recall, F1-score, and the area under the receiver operating characteristic curve (AUC). Precision was also computed because it enters the definition of F1-score, but it was not included as a separate column in the main comparison tables. For a binary task, the pathogen-positive or microbial-marker-present category was treated as the positive class. Precision, recall, and F1-score were defined as Equation 50

$$\begin{array}{l} \text{Precision}=\frac{\text{TP}}{\text{TP}+\text{FP}},\;\;\text{Recall}=\frac{\text{TP}}{\text{TP}+\text{FN}}, \end{array}$$ (50)

and Equation 51

$$\begin{array}{l} \text{F1}=\frac{2\text{Precision}\;\text{Recall}}{\text{Precision}+\text{Recall}}. \end{array}$$ (51)

AUC was defined as the area under the receiver operating characteristic (ROC) curve obtained by varying the classification threshold over the predicted probability. The ROC curve expresses the true-positive rate as a function of the false-positive rate (Equation 52):

$$\begin{array}{l} \text{TPR}=\frac{\text{TP}}{\text{TP}+\text{FN}},\;\;\text{FPR}=\frac{\text{FP}}{\text{FP}+\text{TN}}. \end{array}$$ (52)

For the binary USDA and PAWQ tasks, standard binary ROC-AUC was used. For the five-class Salmonella-serovar and six-class Microcolony tasks, precision, recall, and F1-score were calculated independently for each class using a one-vs-rest formulation and were subsequently macro-averaged. Multiclass AUC was likewise calculated using one-vs-rest ROC curves followed by an unweighted macro-average across classes. Accuracy was calculated as the proportion of correctly classified observations. For datasets containing multiple binary targets, each target was evaluated independently before dataset-level aggregation. USDA summary metrics were obtained by taking the unweighted mean of the Salmonella and Campylobacter target-specific scores, whereas PAWQ summary metrics were obtained by taking the unweighted mean of the four microbial-marker-specific scores. Samples from different prediction targets were not pooled when computing these metrics.

#### 4.2.6 Uncertainty and statistical analysis

The mean and standard deviation across the five repeated experiments describe between-run variation and are reported in the main result tables. Pairwise 95% confidence intervals were additionally estimated for performance differences between methods using 2,000 paired hierarchical bootstrap replicates. For each bootstrap replicate, the random-seed dimension and the original evaluation units were resampled with replacement while preserving the pairing between the two methods being compared. The source hypercube was used as the evaluation unit for the Salmonella hyperspectral dataset, and the establishment was used for the USDA dataset. Individual test images were used for Microcolony, and individual observations were used for PAWQ. For multiclass or binary datasets without a grouping variable, resampling was performed within class strata when required to preserve the observed class structure. For USDA and PAWQ dataset-level summaries, the target-specific metric differences were calculated first and then combined using the same equal weighting used for the reported dataset-level metric. The 2.5th and 97.5th percentiles of the paired bootstrap distribution defined the 95% confidence interval for the performance difference. Statistical comparisons in the main baseline experiments were performed between FSP and the competing method selected by the highest validation F1-score under the same experimental protocol. Ablation analyses used Full FSP as the reference and compared it separately with each corresponding ablated variant. A two-sided paired permutation test with 10,000 permutations was used for both types of comparison. For grouped datasets, predictions were exchanged between the two compared methods at the original group level rather than at the level of derived samples. For ungrouped datasets, the individual observation served as the exchange unit. Repeated predictions of the same evaluation unit across random seeds were kept together during each exchange so that repeated seeds were not treated as independent samples. The test statistic was the mean paired difference in the corresponding evaluation metric across repeated experiments. For multi-target datasets, target-specific differences were calculated before equal-weight aggregation. The family-wise significance level was set to α = 0.05. Holm Bonferroni correction was applied to the reported metric-level comparisons within each dataset. For the ablation analysis, correction was applied jointly across the ablation-versus-Full-FSP comparisons for the reported metrics within the corresponding dataset. A difference is described as statistically significant only when its Holm-adjusted *p*-value is below 0.05; uncorrected mean differences alone are not interpreted as evidence of statistical significance.

### 4.3 Comparison with modality-matched baselines

To ensure technically meaningful comparisons, baseline methods were selected according to the native modality and prediction task of each dataset rather than applying a common set of architectures indiscriminately across all experiments. All methods were evaluated using the same data partitions, random seeds, target definitions, and leakage-control procedures described in Section 4.2. Data-dependent preprocessing operations and hyperparameters were fitted or selected using only the corresponding training and validation partitions. The test partitions were not used for model selection. Therefore, the numerical results reported in this section correspond to implementations evaluated under the experimental protocol of this study rather than values copied directly from the original publications.

For the Salmonella Serovar Hyperspectral Dataset, the comparison includes PCA-kNN, PCA-SVM, PCA-RF, and PCA-MLP, representing conventional dimension-reduction and classification strategies previously evaluated for Salmonella hyperspectral microscopy (Papa et al., 2025). Principal component analysis was fitted independently within each training partition before transforming the corresponding validation and test observations. A one-dimensional convolutional neural network (Kang et al., 2020) and an LSTM-based hyperspectral classifier (Kang et al., 2021) were additionally included because both operate directly on ordered bacterial spectral measurements. These methods provide modality-matched deep-learning comparators for evaluating the contribution of the proposed spectral-consistency and harmonic-relation constraints. For the Microcolony Domain Adaptation Dataset, the source-only comparison includes ResNet-18 (He et al., 2016), EfficientNetV2-S (Tan and Le, 2021), and ConvNeXt-Tiny (Liu et al., 2022). EfficientNetV2 is particularly relevant because this backbone has been used in recent foodborne microcolony classification under acquisition-domain variation (Bhattacharya et al., 2025). A ResNet-18+Context baseline was also constructed using the same acquisition metadata supplied to FSP. In this baseline, the encoded metadata are concatenated directly with the image representation before classification, providing a control for whether performance differences arise simply from access to contextual information or from the gated contextual representation used by FSP. Domain-adversarial methods requiring labeled or unlabeled target-domain samples during adaptation were not included in the principal comparison because the experiments reported here follow a source-only training protocol. For the USDA Raw Poultry and PAWQ Agricultural Water datasets, the comparison covers conventional tree ensembles and recent neural methods for structured data. The evaluated models include Random Forest (Breiman, 2001), XGBoost (Chen and Guestrin, 2016), CatBoost (Prokhorenkova et al., 2018), FT-Transformer (Gorishniy et al., 2021), TabR (Gorishniy et al., 2024), and TabM (Gorishniy et al., 2025). Each tabular baseline received the union of the primary and contextual predictor variables available to the proposed framework. Consequently, differences between FSP and the tabular baselines reflect representation and modeling choices rather than access to additional predictive covariates.

Table 2 reports the results for the hyperspectral and microscopy datasets. On Salmonella serovar classification, the PCA-based models provide useful conventional references, while the deep spectral classifiers obtain stronger results on the ordered wavelength measurements. Among the comparison methods, 1D-CNN achieves the highest baseline accuracy, macro-recall, macro-F1, and macro-AUC. FSP increases mean accuracy from 82.66 to 84.91%, macro-F1 from 81.59 to 83.97%, and macro-AUC from 94.08 to 95.31%. The corresponding macro-recall increases from 81.87 to 84.26%. These descriptive differences are consistent with the intended role of the spectral-consistency and harmonic-relation objectives, which encourage the latent representation to retain information associated with the native wavelength structure. The Microcolony results exhibit a different pattern. Under the standard-domain test condition, EfficientNetV2-S obtains the highest mean accuracy (95.74%), whereas FSP obtains the highest mean F1 (95.34%) and AUC (98.87%). Thus, the proposed method does not dominate every metric when training and test acquisition conditions are closely matched. Larger differences are observed on the predefined OOD test set. FSP reaches an OOD accuracy of 67.43%, F1 of 65.92%, and AUC of 84.11%. The corresponding values for EfficientNetV2-S are 62.37, 60.88, and 81.26%, respectively. The ResNet-18+Context baseline narrows this gap, reaching an OOD F1 of 63.74% and an AUC of 82.73%. This comparison indicates that acquisition metadata contain useful information under domain shift and that the gated contextual formulation provides additional improvement over simple late concatenation. Because no target-domain adaptation is performed in these experiments, the results should not be interpreted as a comparison with methods operating under an explicit domain-adaptation protocol.

Table 3 reports the results for the two structured-data benchmarks. On the USDA Raw Poultry Dataset, the gradient-boosted tree methods remain competitive, while the more recent tabular neural models reduce the gap relative to FSP. Among the baselines, TabM provides the strongest overall results, with an accuracy of 86.62%, recall of 74.13%, F1 of 73.46%, and AUC of 89.97%. FSP obtains corresponding mean values of 87.18%, 75.36%, 74.61%, and 90.57%. The largest absolute difference is observed in recall, which is particularly relevant in this setting because pathogen-positive observations constitute the positive class and overall accuracy alone does not describe positive-case sensitivity. The PAWQ experiment illustrates a different behavior. TabM achieves the highest mean accuracy at 78.79%, compared with 78.63% for FSP. Conversely, FSP obtains the highest mean recall (68.74%), F1 (67.96%), and AUC (81.11%). The differences between several of the stronger structured-data models are smaller than those observed for the hyperspectral and Microcolony OOD experiments, and between-run variability is also larger. The PAWQ results therefore provide a useful counterexample to a claim of universal superiority: the proposed representation improves several positive-class and ranking metrics while the strongest tabular baseline remains competitive in overall accuracy.

**Table 3:** Comparison with tree-based and neural tabular methods on the USDA Raw Poultry and PAWQ Agricultural Water datasets.

Model	Accuracy	Recall	F1 Score	AUC
**(a) USDA Raw Poultry Salmonella Campylobacter Dataset**				
Random Forest (Breiman, 2001)	83.77 ± 0.48	68.54 ± 1.08	67.31 ± 0.96	85.92 ± 0.55
XGBoost (Chen and Guestrin, 2016)	85.26 ± 0.41	71.63 ± 0.92	70.48 ± 0.84	88.14 ± 0.47
CatBoost (Prokhorenkova et al., 2018)	85.71 ± 0.45	72.21 ± 0.87	71.34 ± 0.81	88.76 ± 0.43
FT-Transformer (Gorishniy et al., 2021)	86.03 ± 0.39	73.08 ± 0.85	72.27 ± 0.79	89.21 ± 0.40
TabR (Gorishniy et al., 2024)	86.21 ± 0.43	73.46 ± 0.81	72.84 ± 0.76	89.58 ± 0.38
TabM (Gorishniy et al., 2025)	86.62 ± 0.37	74.13 ± 0.80	73.46 ± 0.74	89.97 ± 0.36
**FSP**	**87.18 ± 0.35**	**75.36 ± 0.78**	**74.61 ± 0.73**	**90.57 ± 0.34**
**(b) PAWQ Agricultural Water Dataset**				
Random Forest (Breiman, 2001)	75.38 ± 1.74	64.89 ± 2.47	64.21 ± 2.32	77.63 ± 1.61
XGBoost (Chen and Guestrin, 2016)	76.91 ± 1.63	66.57 ± 2.29	65.84 ± 2.18	79.18 ± 1.48
CatBoost (Prokhorenkova et al., 2018)	78.24 ± 1.58	66.95 ± 2.23	66.41 ± 2.09	80.32 ± 1.44
FT-Transformer (Gorishniy et al., 2021)	74.81 ± 1.79	64.12 ± 2.55	63.38 ± 2.37	77.09 ± 1.66
TabR (Gorishniy et al., 2024)	76.66 ± 1.69	65.83 ± 2.36	65.17 ± 2.21	79.42 ± 1.53
TabM (Gorishniy et al., 2025)	**78.79 ± 1.52**	67.43 ± 2.14	67.05 ± 2.01	80.88 ± 1.39
**FSP**	78.63 ± 1.49	**68.74 ± 2.06**	**67.96 ± 1.93**	**81.11 ± 1.35**

Values are percentages and are reported as mean ± standard deviation over five independent random seeds. USDA results are the unweighted averages of the Salmonella and Campylobacter target-specific metrics, whereas PAWQ results are the unweighted averages of the four microbial-source-marker tasks. Boldface indicates the highest mean value in each column and does not by itself denote statistical significance.

Taken together, the descriptive results do not indicate that a single architecture dominates every metric under all sensing conditions. The largest mean differences are observed in settings where the FSP formulation can use additional structure that is native to the corresponding dataset. On the Salmonella hyperspectral task, spectral-consistency and harmonic information are available; on USDA, contextual meteorological information and valid temporal predecessor target relations can be incorporated; and on the Microcolony benchmark, acquisition context is available for OOD prediction. PAWQ provides a more conservative case in which contextual information is available but the small structured dataset leaves recent tabular baselines highly competitive. The inferential procedure for pairwise method comparison is defined in Section 4.2. The mean and standard deviation values in Tables 2, 3 are interpreted descriptively, and typographic emphasis identifies only the highest observed mean. No difference is described as statistically significant on the basis of these descriptive statistics alone.

### 4.4 Ablation study

The ablation experiments were designed according to the information sources actually available in each dataset. Because FSP conditionally activates contextual, temporal, and spectral mechanisms, removing a component that is inactive for a given dataset would not provide a meaningful estimate of its contribution. The spectral and harmonic components are therefore examined on the Salmonella hyperspectral dataset, the temporal mechanism is examined on the USDA Raw Poultry dataset, and contextual representation is examined on the Microcolony and PAWQ datasets. “Full FSP” denotes the complete configuration containing all components applicable to the corresponding dataset. All ablation variants use the same preprocessing, data partitions, random seeds, optimization settings, predictor variables, and evaluation protocol as the complete configuration. When an auxiliary objective is removed, the remaining architecture and training settings are unchanged. When an evidence branch is removed, all other branches retain exactly the same inputs as in Full FSP. Variants that modify contextual fusion likewise retain the same underlying contextual variables unless context removal is explicitly part of the ablation. This design reduces confounding from differences in data access, training procedure, or evaluation protocol.

Table 3 reports ablations for the Salmonella hyperspectral and USDA Raw Poultry datasets. For the hyperspectral task, four variants are considered. The first removes the direct spectral-consistency objective $L_{\text{spec}}$, while the second removes the harmonic-relation objective $L_{\text{harm}}$. The third disables the complete spectral-evidence path, including the generation of ${\tilde{q}}_{j}$ and its transformation into latent spectral evidence **s**_*j*_. The fourth retains both current and spectral representations but replaces the learned availability-aware mixture weights with equal weights. These variants separately examine coefficient-level spectral supervision, harmonic-relation regularization, the spectral evidence representation itself, and adaptive evidence integration. Removing the complete spectral-evidence path produces the largest observed decrease on the Salmonella task, with macro-F1 changing from 83.97 to 81.64% and macro-AUC from 95.31 to 93.72%. Removing $L_{\text{spec}}$ results in a larger mean reduction than removing only $L_{\text{harm}}$, which is consistent with the direct spectral objective providing the primary coefficient-level constraint and the harmonic objective acting as complementary relation-level regularization. Equal-weight evidence fusion also produces lower mean performance than the learned integrator, suggesting that the contribution of latent spectral evidence need not be constant across observations. For the USDA dataset, the applicable auxiliary mechanism is temporal rather than spectral. The temporal-branch ablation removes the forward-projected representation ${\tilde{h}}_{j}$ entirely, whereas the “w/o $L_{\text{temp}}$” variant retains the temporal branch but removes its projection-consistency supervision. A separate context-removal variant suppresses meteorological context while retaining the primary structured predictors. Uniform evidence fusion replaces the learned mixture weights between the current state and temporally aligned evidence with equal weights. Among these variants, context removal produces the largest observed decrease in recall, while removal of the complete temporal branch reduces both F1 and AUC relative to Full FSP. Retaining the temporal branch without $L_{\text{temp}}$ yields an intermediate result, which is consistent with complementary contributions from the projected representation and its temporal-consistency constraint.

Table 5 examines the Microcolony and PAWQ datasets, for which the temporal and spectral auxiliary branches are disabled. The ablations therefore focus on whether contextual variables contribute useful information and whether separating primary and contextual representations before gated fusion is preferable to simpler alternatives. For the Microcolony dataset, the ablation analysis is reported on the predefined OOD test set because acquisition metadata are most relevant when the image-acquisition conditions differ from the source-training domain. The image-only variant removes the contextual acquisition descriptors and uses only the microscopy representation. The late-concatenation variant retains the same metadata available to Full FSP but replaces gated residual fusion with direct concatenation before prediction. The image-only configuration shows substantially lower mean OOD performance than the context-aware variants. Restoring the same metadata through late concatenation recovers much of this difference, while Full FSP further increases mean accuracy, recall, F1, and AUC. This pattern is consistent with both the contextual variables themselves and the gated representation mechanism contributing to robustness under acquisition shift. For PAWQ, three contextual variants are considered. “Primary variables only” removes weather, land-cover, livestock-proximity, and infrastructure context while retaining the water-quality and non-target microbiological predictors. “Single tabular encoder” provides the union of primary and contextual predictors to one encoder, eliminating the explicit distinction between their representational roles. “Late concatenation” preserves separate primary and contextual encoders but replaces gated residual fusion with direct concatenation. Full FSP obtains the highest observed mean recall, F1, and AUC, while its advantage over late concatenation is smaller than the difference between the context-free and context-aware configurations. This pattern is consistent with environmental context providing complementary predictive information, with an additional but more modest contribution from the gated fusion design.

The ablation results support an availability-aware interpretation of FSP rather than the assumption that every component contributes uniformly to every dataset. For hyperspectral observations, the direct spectral constraint, harmonic-relation regularization, and latent spectral-evidence pathway provide distinct contributions. For temporally linked structured observations, the forward-projected representation and its consistency objective provide complementary temporal information. For microscopy and environmental tabular data, contextual variables and their representation strategy affect performance even when no temporal or spectral auxiliary branch is active. These observations are consistent with the conditional formulation developed in Sections 3.2-3.4. The inferential procedure for ablation comparisons is defined in Section 4.2, where each ablated configuration is paired with Full FSP under identical splits and random seeds. The values in Tables 4, 5 are therefore interpreted here as descriptive mean differences unless a Holm-adjusted statistical comparison is explicitly reported.

**Table 4:** Ablation of spectral and temporal components on datasets for which the corresponding auxiliary information is available.

Variant	Accuracy	Recall	F1 score	AUC
**(a) Salmonella Serovar Hyperspectral Dataset**				
w/o $L_{\text{spec}}$	83.88 ± 0.55	83.24 ± 0.69	82.93 ± 0.64	94.51 ± 0.43
w/o $L_{\text{harm}}$	84.31 ± 0.52	83.71 ± 0.65	83.40 ± 0.60	94.84 ± 0.40
w/o spectral evidence branch	82.79 ± 0.62	82.02 ± 0.77	81.64 ± 0.71	93.72 ± 0.49
Uniform evidence fusion	84.17 ± 0.50	83.55 ± 0.62	83.29 ± 0.58	94.68 ± 0.39
**Full FSP**	**84.91 ± 0.47**	**84.26 ± 0.58**	**83.97 ± 0.55**	**95.31 ± 0.36**
**(b) USDA Raw Poultry Salmonella Campylobacter Dataset**				
w/o temporal evidence branch	86.44 ± 0.42	73.19 ± 0.96	72.47 ± 0.90	89.62 ± 0.42
w/o $L_{\text{temp}}$	86.79 ± 0.39	74.37 ± 0.88	73.66 ± 0.83	90.06 ± 0.38
w/o context	86.31 ± 0.46	72.88 ± 1.02	72.15 ± 0.94	89.41 ± 0.45
Uniform evidence fusion	86.86 ± 0.38	74.58 ± 0.85	73.89 ± 0.79	90.15 ± 0.37
**Full FSP**	**87.18 ± 0.35**	**75.36 ± 0.78**	**74.61 ± 0.73**	**90.57 ± 0.34**

Values are percentages reported as mean ± standard deviation over five independent random seeds. “Full FSP” includes all components applicable to the corresponding dataset. Boldface denotes the highest observed mean and does not by itself indicate statistical significance.

**Table 5:** Ablation of contextual representation and fusion on the Microcolony and PAWQ datasets.

Variant	Accuracy	Recall	F1 score	AUC
**(a) Microcolony Domain Ddaptation Dataset OOD Test**				
Image only (w/o context)	55.02 ± 1.76	54.11 ± 2.01	53.28 ± 1.93	76.11 ± 1.18
Late concatenation	65.21 ± 1.36	64.33 ± 1.67	63.74 ± 1.52	82.73 ± 0.81
**Full FSP**	**67.43 ± 1.29**	**66.71 ± 1.57**	**65.92 ± 1.44**	**84.11 ± 0.76**
**(b) PAWQ Agricultural Water Dataset**				
Primary variables only	75.92 ± 1.81	65.15 ± 2.48	64.38 ± 2.31	78.24 ± 1.62
Single tabular encoder	77.52 ± 1.67	66.83 ± 2.29	66.09 ± 2.16	79.84 ± 1.50
Late concatenation	78.06 ± 1.57	67.45 ± 2.18	66.73 ± 2.04	80.39 ± 1.42
**Full FSP**	**78.63 ± 1.49**	**68.74 ± 2.06**	**67.96 ± 1.93**	**81.11 ± 1.35**

Values are percentages reported as mean ± standard deviation over five independent random seeds. Microcolony results correspond to the predefined OOD test set, and PAWQ metrics are unweighted averages across the four microbial-source-marker prediction tasks. Boldface denotes the highest observed mean and does not by itself indicate statistical significance.

## 5 Conclusions and future work

This study presented the ForwardSpectralProbabilistic (FSP) framework for microbial classification and pathogen-risk prediction under heterogeneous food-safety monitoring conditions. Rather than assuming that all observations share the same sensing modality or auxiliary information, FSP employs modality-specific encoders for hyperspectral, microscopy-image, and structured observations and maps their outputs to a common *d*-dimensional latent interface. The datasets are trained and evaluated independently, while the same availability-aware formulation is maintained across tasks. Contextual variables are incorporated through gated residual fusion when environmental, operational, or acquisition information is available. The Forward Consistency Projector produces temporally aligned latent evidence only for observations with a valid predecessor target correspondence, whereas the Spectral Consistency Mapper and harmonic-relation constraint are restricted to measurements with a native spectral axis. The Probabilistic Mixture Integrator then combines only the current, temporal, and spectral representations that are available for an observation before a dataset-specific prediction head produces the final class or pathogen-risk probability. The experimental evaluation considered four heterogeneous microbial-safety benchmarks covering Salmonella serovar classification from hyperspectral measurements, microscopy-based bacterial classification, poultry pathogen-risk prediction, and agricultural-water microbial-marker prediction. The descriptive results show that FSP is competitive with modality-matched conventional and recent learning methods, although the strongest observed performance is not obtained by FSP for every metric and dataset. The hyperspectral experiment shows the clearest benefit from spectral-consistency and harmonic-relation modeling, while the USDA experiment indicates that meteorological context and temporally aligned evidence can contribute to pathogen-risk prediction. On the Microcolony benchmark, acquisition metadata are particularly useful under out-of-distribution conditions, whereas the PAWQ results show more modest differences among the stronger structured-data models. The ablation experiments further support an availability-aware interpretation of the framework: spectral, temporal, and contextual mechanisms contribute only in settings where the corresponding information source is present.

Several limitations should be considered when interpreting these results. The four datasets represent different prediction tasks, sensing modalities, sample sizes, and label definitions. Their heterogeneity is useful for evaluating whether the same modeling formulation can accommodate different information structures, but it should not be interpreted as an end-to-end evaluation of a single fresh-food supply chain. In particular, the available datasets do not provide complete longitudinal trajectories linking production, processing, transportation, storage, and retail observations for the same product or batch. The temporal mechanism is therefore evaluated only on observations for which a defensible predecessor target relation can be constructed, rather than on complete product-level microbial trajectories. The scale and distributional characteristics of the datasets vary substantially. The agricultural-water benchmark contains comparatively few observations, while the microscopy benchmark includes acquisition shifts that produce considerable changes between source and OOD conditions. These characteristics increase uncertainty in performance estimates and limit the extent to which the reported results can be generalized directly to unseen regions, facilities, products, laboratories, or sensing platforms. Grouped or stratified evaluation, repeated random seeds, and explicit OOD testing reduce some sources of evaluation bias, but independent external validation remains necessary before operational deployment. This study focuses on predictive performance, representation design, and component-level behavior rather than computational deployment cost. Modality-specific encoders, auxiliary consistency objectives, and conditional evidence pathways may introduce additional training or inference requirements relative to simpler single-modality models. Because latency, memory consumption, parameter count, and computational overhead were not evaluated as dedicated experimental outcomes, no claim of improved computational efficiency is made.

Future work will focus on three directions. Genuinely longitudinal microbial-safety datasets are needed in which microbial measurements, environmental conditions, handling events, and supply-chain stages are linked at the level of the same product, batch, or other defensible tracking unit. Such datasets would permit the Forward Consistency Projector to be evaluated over longer and more realistic temporal trajectories. External validation and cross-domain generalization should be investigated across geographic regions, food categories, production facilities, laboratories, imaging systems, and sensing platforms. Domain adaptation, probability calibration, uncertainty estimation, and robustness to partially missing context are particularly relevant extensions. Deployment-oriented evaluation should examine model size, inference latency, memory consumption, and energy or hardware requirements, together with lightweight encoders, conditional branch pruning, and more efficient evidence integration. The principal contribution of FSP is an availability-aware modeling strategy that does not require temporal, spectral, image, and contextual information to be simultaneously present. Instead, each information source is used only when it is supported by the corresponding observation, while the prediction process remains expressed through a consistent latent representation and evidence-integration framework. This formulation provides a basis for developing microbial-monitoring systems that must operate across heterogeneous sensing and food-safety scenarios without imposing artificial modalities or temporal relationships on the underlying data.

## Data Availability

The original contributions presented in the study are included in the article/supplementary material, further inquiries can be directed to the corresponding author.
